# Impella Versus Selective Biatrial Canulation for Left Ventricular Unloading During Extracorporeal Membrane Oxygenation

**DOI:** 10.1155/cdr/3669575

**Published:** 2026-02-08

**Authors:** Mouhamed Djahoum Moussa, Jean Roux, Marie Jungling, Nassima Ramdane, Valentin Loobuyck, Benoit Brassart, Natacha Rousse, Céline Dupré, Agnès Mugnier, Abdelilah Khalipha, Adham Sameer A. Bardeesi, Oliver Lukowiak, Loïc Lefebvre, Francis Juthier, Emmanuel Robin, Julien Labreuche, Lise Thellier, André Vincentelli

**Affiliations:** ^1^ CHU Lille, Pôle d′Anesthésie-Réanimation, Lille, France, chru-lille.fr; ^2^ Univ. Lille, ULR 2694-METRICS: évaluation des technologies de santé et des pratiques médicales, CHU Lille, Lille, France, chru-lille.fr; ^3^ CHU Lille, Department of Cardiac Surgery, Lille, France, chru-lille.fr; ^4^ CHU Lille, Department of Biostatistics, Lille, France, chru-lille.fr

**Keywords:** cannulated atrioseptostomy, Impella, left ventricular distension, left ventricular unloading, outcomes, selective biatrial ECMO, venoarterial extracorporeal membrane oxygenation

## Abstract

**Objectives:**

Comparisons of preload unloading techniques for left ventricle overdistension during venoarterial (VA) extracorporeal membrane oxygenation (ECMO) support are scarce. We compared outcomes in patients with left ventricular distension treated with cannulated percutaneous atrioseptostomy combined with ECMO—specified as selective biatrial extracorporeal membrane oxygenation (SBA‐ECMO)—versus those treated with Impella CP/5.0 in combination with ECMO (ECPELLA).

**Methods:**

Consecutive adult patients who received VA‐ECMO and underwent additional left ventricle unloading between January 2014 and March 2023 were studied. The primary endpoint was the number of ventilation‐free days. The secondary endpoints were serious bleeding, blood product consumption, thrombotic complications, and 28‐day mortality.

**Results:**

We included 57 patients, 27 of whom received SBA‐ECMO and 30 of whom received ECPELLA. The median number of ventilation‐free days was 10 days (0–23) with SBA‐ECMO and 5 days (0–23) with the ECPELLA (*p* = 0.61). According to the multivariable analyses, SBA‐ECMO was associated with a lower risk of serious bleeding (HR 0.31 [95% CI 0.12–0.80]) and less blood product consumption (RR 0.57 [95% CI 0.36–0.90]) than ECPELLA. Thrombotic complications and 28‐day mortality were similar between the groups before and after multivariable analyses.

**Conclusions:**

In patients with left ventricle congestion during VA‐ECMO support, left ventricle unloading with SBA‐ECMO was associated with reduced serious bleeding and transfusions compared with ECPELLA, despite a similar number of ventilation‐free days, thrombotic complications, and mortality.

**Trial Registration:**

ClinicalTrials.gov identifiers: NCT03431467 and NCT05577195

## 1. Introduction

Venoarterial extracorporeal membrane oxygenation (VA‐ECMO) is the mainstay of circulatory support for refractory cardiogenic shock [1, 2]. However, VA‐ECMO imposes an increased afterload on the left ventricle (LV), which may favor LV distention with subsequent poor subendocardial perfusion, pulmonary edema, and compromised cardiac recovery [3–5].

LV unloading is an appropriate response to this complication, yet a growing body of literature suggests that prophylactic or early unloading may further improve outcomes during VA‐ECMO support [6–9]. A major question is which type of unloading strategy should be prioritized. Two main approaches to LV unloading can be considered: the preload reduction approach, which has been shown to be superior to the LV afterload reduction approach, namely, the intra‐aortic balloon pump (IABP) [5]. The classical LV preload reduction approach includes LV apical cannulation and pulmonary vein cannulation, both of which require a ministernotomy or a thoracotomy. Less invasive preload unloading techniques that are performed percutaneously or through a limited surgical incision are also available. These methods mainly include transaortic drainage with an Impella axial pump (Abiomed Inc., Danvers, MA), pulmonary artery venting, classical balloon atrioseptostomy, and its upgraded version, the cannulated atrioseptostomy (CAST), which allows biatrial drainage and is called selective biatrial ECMO (SBA‐ECMO) [10–13]. SBA‐ECMO and the Impella combined with VA‐ECMO (ECPELLA) are the most recent and studied [9, 12, 14]. Compared with the noncanulated approach, SBA‐ECMO has been shown to be effective and consistent in LV unloading and has a good safety profile [10–12, 14]. ECPELLA has also been shown to be effective for LV unloading, with improved outcomes [9].

The performance of these two relevant preload unloading techniques (ECPELLA and SBA‐ECMO) has not yet been compared. The aim of this study was to compare the clinical outcomes associated with these two techniques in patients who develop LV distension. Our primary objective was to compare their effects on ventilation‐free days (VFDs), and our secondary objectives were to compare their effects on blood product consumption (BPC), serious bleeding, thrombotic complications, and 28‐day mortality.

## 2. Materials and Methods

### 2.1. Ethics

This retrospective study was conducted in the academic cardiovascular intensive care unit (ICU) of Lille University Hospitals, a referral center for ECMO.

The Ethical Committee of the French Society of Anesthesia and Intensive Care Medicine approved the study in September 2021 (IRB 00010254‐2021‐169) and waived the need for informed consent due to the retrospective design. The datasets used were declared to the French authorities (CNIL) in accordance with French laws.

### 2.2. Patients

We included adult patients (> 18 years) who were supported by VA‐ECMO between January 2014 and March 2023 for refractory cardiogenic shock and who received additional LV unloading for symptomatic LV distension (pulmonary edema, LV sludge, and persistent aortic valve closure despite ECMO flow lowering with pulmonary capillary wedge pressure [PCWP] > 20 mmHg). Only the first ECMO support is considered in case of multiple ECMO runs; the other exclusion criteria were right ventricle‐to‐pulmonary artery VA‐ECMO and any other LV unloading approach that SBA‐ECMO or ECPELLA.

### 2.3. Data Collection

Clinical, biological, radiological, and prognostic data were collected from physical and electronic clinical records (Sillage [SIB, Rennes, France] and IntelliSpace Critical Care and Anesthesia [Philips Healthcare, Koninklijke Philips N.V., the Netherlands]). Patients′ daily chest radiographs were retrospectively analyzed for the occurrence of pulmonary edema and the presence of an endotracheal tube to refine the number of days without invasive mechanical ventilation.

We collected baseline anthropometric data, comorbidities, the LV ejection fraction, the type of cannulation, the etiology of refractory cardiogenic shock, and the history of cardiac arrest before VA‐ECMO placement. We also collected arterial lactate levels, the biological data needed to calculate the Simplified Acute Physiologic Score II (SAPS II), and those needed to define study outcomes.

### 2.4. Transfusion and Bleeding Management

The transfusion and bleeding management are based on predefined local protocols. Unfractionated heparin (UFH) was used unless contraindicated, with a bolus of 50–100 IU/kg given before cannulations, followed by an intravenous infusion to obtain an anti‐FXa of 0.2–0.7 IU/mL. For the Impella devices, a heparinized purge solution was used according to the manufacturer′s recommendation with an additional systemic anticoagulation, both adjusted to obtain a similar target as abovementioned. Targets were adjusted according to the pump flows, the occurrence of bleeding or thrombotic complications, and the underlying disease. Anticoagulation‐free support may be considered at the physician′s discretion in cases of overt bleeding. Hemostasis was managed in ICUs using a multiparametric approach based mainly on aPTT, PT, anti‐FXa, fibrinogen, platelet count, and, since 2019, thromboelastography.

Packed red blood cells (PRBCs) were transfused to target a hemoglobin level between 7 and 9 and a ScvO_2_ ≥ 65*%*, and platelet concentrates were used to obtain a platelet count > 50,000 mm^−3^ (increased to 70,000 mm^−3^ in case of bleeding). Fresh frozen plasma was suggested for patients who experienced overt bleeding and who required massive transfusion with a predefined FFP:RBC ratio, according to multiparametric assessment of coagulation tests (PT, aPTT, fibrinogen, and thromboelastography).

### 2.5. LV Unloading Techniques

LV unloading was considered in the presence of LV distension with spontaneous contrast with a closed aortic valve with nonpulsatile flow or pulmonary edema. The full description of our LV unloading strategy is provided in the supporting information methods and has been published in part elsewhere [15]. LV unloading was performed either with an Impella CP/5.0 (Abiomed Inc., Danvers, MA) or with the SBA‐ECMO configuration after multidisciplinary discussion involving the referral cardiac surgeon, cardiologist, and intensivist. The main criteria were technical feasibility for each approach and the presence of a contraindication for either of the two techniques.

The Impella CP was placed percutaneously and positioned across the aortic valve under fluoroscopy and transesophageal echocardiography (TOE) guidance. For Impella 5.0, the device was inserted through a Dacron prosthesis sutured to the right subclavian artery and positioned under fluoroscopy and TOE guidance.

**Figure 1:**
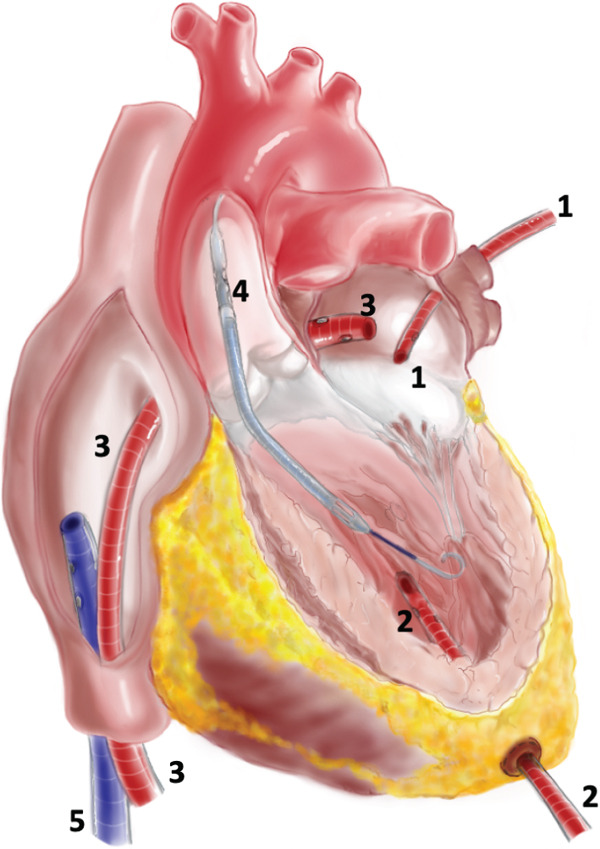
Preload left ventricular unloading approaches. (1) Pulmonary vein left ventricle (LV) venting. (2) Transapical LV venting. (3) Transseptal canula achieved after atrioseptostomia, which is associated with five results in selective biatrial ECMO (SBA‐ECMO). (4) Impella CP/5.0, which is on top of ECMO results in ECPELLA. (5) Usual venous drainage cannula.

To achieve SBA‐ECMO, CAST was performed under TOE and fluoroscopic guidance with an additional drainage cannula placed in the left atrium. This cannula was inserted percutaneously into the femoral vein opposite to the initial venous cannula to drain the left atrium. Both drainage cannulas were connected (via a 3/8 ^″^ Y connector) before reaching the ECMO pump head inflow (Figure 1 and Figure S1).

### 2.6. Study Outcomes

The primary outcome was VFDs, defined as the number of days alive without invasive mechanical ventilation from LV unloading until 28 days or hospital discharge.

All secondary outcomes were assessed from LV unloading until 28 days or hospital discharge and were as follows:

i. Serious bleeding was defined according to the ELSO bleeding criteria as bleeding requiring surgical exploration or characterized by its location (central nervous system, hemothorax, and retroperitoneal bleeding) or requiring immediate transfusion of at least 2 units of PRBC for either a sudden decrease in hemoglobin of 2 g/dL in less than 24 h or new onset of hemodynamic instability or overt bleeding.
ii. Thrombotic complications, a composite of stroke, limb ischemia, aortic root or intracardiac thrombosis, mesenteric ischemia, and ECMO, or any thrombosis that led to medical or surgical intervention or death.
iii. BPC was defined as the cumulative number of PRBCs, platelets, and fresh frozen plasma transfused from LV unloading to the end of VA‐ECMO support.
iv. Twenty‐eight‐day mortality was defined as all‐cause mortality from LV unloading to 28 days.

### 2.7. Statistical Analysis

Categorical variables are expressed as numbers (percentages). Quantitative variables are expressed as means (standard deviations) if normally distributed or as medians (interquartile ranges) otherwise. The normality of the distribution was assessed using histograms and the Shapiro–Wilk test.

Patient characteristics at the time of LV unloading were described and compared between groups using a chi‐square test or Fisher′s exact test for categorical variables, the Mann–Whitney test for non‐Gaussian quantitative variables, and Student′s *t* test for Gaussian quantitative variables. Comparisons between the two study groups on the primary outcome (VFDs) were performed using the Mann–Whitney test and by using nonparametric analysis of covariance to account for the predefined confounders. BPC during LV unloading was compared between the two study groups using a negative binomial regression model (rate ratios [RRs] with 95% confidence intervals [CIs] as the effect size) with ECMO duration from LV unloading as the offset variable before and after adjustment for predefined confounders. The cumulative incidence of bleeding and thrombotic events during ECMO was estimated using the Kalbfleisch and Prentice approach by considering death as a competing event. Between‐group comparisons were made using the cause‐specific hazard ratios (cHRs) with their 95% CIs derived from Cox proportional hazard regression models before and after adjustment for predefined confounders. The all‐cause mortality rate at 28 days was compared between the study groups using a logistic regression model (odds ratios [ORs] with 95% CIs as the effect size) before and after adjustment for predefined confounders. The predefined confounders were SAPS II, postcardiotomy, and time from ECMO to LV unloading.

Statistical tests were performed at a two‐tailed *α* level of 0.05. The data were analyzed using the SAS software package, Version 9.4 (SAS Institute, Cary, NC, United States).

## 3. Results

**Figure 2:**
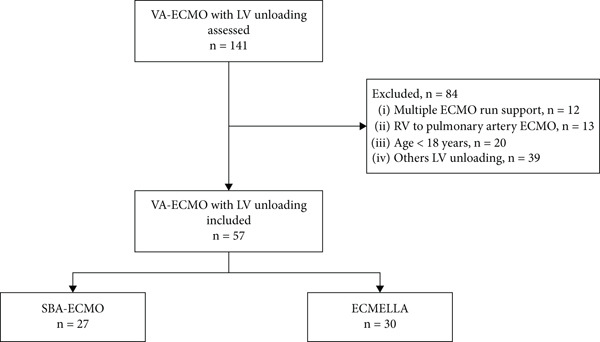
Study flowchart. VA‐ECMO, venoarterial extracorporeal membrane oxygenation; LV, left ventricle; SBA‐ECMO, selective biatrial ECMO; ECPELLA, Impella CP/5.0 on top of ECMO.

Of the 141 patients who underwent VA‐ECMO with LV unloading during the study period, 57 patients were included—27 (47.4%) and 30 (52.6%) in the SBA‐ECMO and ECPELLA groups, respectively. All patients suffered pulmonary edema on the day of unloading and were mechanically ventilated. Of those, 54 (94.7%) patients were invasively ventilated. The study flowchart is shown in Figure 2. The rate of history of ischemic heart disease and baseline lactate were different between groups, and the other comorbidities were comparable (Table 1). Figure S2 shows a trend toward higher levels of anti‐FXa in the ECPELLA group compared to the SBA‐ECMO group.

**Table 1:** Patient characteristics according to the studied left ventricle unloading groups.

**Variables**	**SBA-ECMO (** **N** = 27**)**	**ECMELLA (** **N** = 30**)**	**p** **value**
Comorbidities			
Age (years)	49 (43–58)	57 (38–64)	0.26
Male gender	18 (66.7)	25 (83.3)	0.14
Body mass index (kg/m^2^)	27.2 ± 6.8	26.6 ± 4.7	0.70
History of stroke	2 (7.4)	4 (13.3)	NA
Atrial fibrillation	7 (25.9)	7 (23.3)	0.82
Diabetes mellitus	6 (22.2)	8 (26.7)	0.70
Hypertension	7 (25.9)	15 (50.0)	0.062
Dyslipidemia	6 (22.2)	9 (30.0)	0.51
eGFR < 60 (mL/min/m^2^)	5 (18.5)	10 (33.3)	0.20
Ischemic heart disease	9 (33.3)	18 (60.0)	0.044
Valvular heart disease	6 (22.2)	6 (20.0)	0.84
Prognostic variables			
Cardiac arrest before ECMO	11 (40.7)	14 (46.7)	0.65
SAPS II	66.6 ± 17.2	59.5 ± 20.2	0.16
LV ejection fraction (%)	12.5 (5–20)	10 (0–15)	0.23
Lactate (mmol/L)	5.06 (2.5–9.49)	2.68 (1.6–4.6)	0.008
ECMO support			
Type of cannulation			
Femorofemoral	23 (85.2)	24 (80.0)	0.73
Axillofemoral	4 (14.8)	6 (20.0)	
Etiology of support			NA
LCOS	3 (11.1)	3 (10.0)	
Heart transplant	0 (0)	3 (10)	
Myocardial infarction	15 (55.6)	13 (43.3)	
Chronic heart disease	6 (22.2)	9 (30)	
Myocarditis	2 (7.4)	1 (3.3)	
Others	1 (3.7)	1 (3.3)	

*Note:* Values are mean ± standard deviation, median (interquartile range, 25^e^–75^e^), or number (percentage).

Abbreviations: eGFR, estimated glomerular filtration rate; LCOS, low cardiac output syndrome; LV, left ventricle; SAPS II, Simplified Acute Physiologic Score II.

### 3.1. VFDs

The median VFDs (IQRs) of the SBA‐ECMO and ECPELLA groups were not significantly different, 10 days (0–23) and 5 days (0–23), respectively (*p* = 0.61) (Table 2). After adjusting for predefined confounders, the difference remained nonsignificant (*p* = 0.22).

**Table 2:** Comparison of main study outcomes in univariable analyses.

**Variables**	**SBA-ECMO (** **N** = 27**)**	**ECPELLA (** **N** = 30**)**	**p** **value**
Ventilation‐free days (days)	10 (0–23)	5 (0–23)	0.61
Blood product transfusion (units)	6 (3–25)	19 (9–27)	0.017
Serious bleeding, *n* (%)	7 (26)	19 (63)	0.007
Serious bleeding types, *n* (%)			
Access or cannulation site bleeding	2 (7)	10 (33)	
Pericardial or pleural bleeding	2 (7)	5 (17)	
Gastrointestinal bleeding	2 (7)	2 (7)	
Intracerebral hemorrhage	1 (4)	1 (3)	
Otorhinolaryngological bleeding	0	1 (3)	
Thrombotic complications, *n* (%)	6 (22)	11 (37)	0.297
Type of thrombotic complications, *n* (%)			
Ischemic stroke	3 (11)	3 (10)	
Limb ischemia	3 (11)	3 (10)	
Anoxo‐ischemic brain lesions	0	4 (13)	
Mesenteric ischemia	0	1 (0)	
Intracardiac thrombus	0	0	
28‐day mortality, *n* (%)	13 (48)	14 (47)	0.91

*Note:* Ventilation‐free days, number of days alive without mechanical ventilation.

Abbreviations: ECPELLA, LV unloading by Impella CP/5.0 on top of VA‐ECMO; SBA, selective biatrial ECMO achieved using cannulated percutaneous atrioseptostomy.

### 3.2. BPC

The overall cumulative incidence rate of BPC was 1.62 units/day (95% CI 1.15–2.27) in the SBA‐ECMO group and 2.84 units/day (95% CI 2.07–3.90) in the ECPELLA group (*p* = 0.017) (Table 2). Compared with ECPELLA, SBA‐ECMO was associated with reduced BPC before (RR 0.57 [95% CI 0.36–0.90], *p* = 0.017) and after adjustment for confounders (RR 0.54 [95% CI 0.34–0.85], *p* = 0.009).

### 3.3. Serious Bleeding

**Figure 3:**
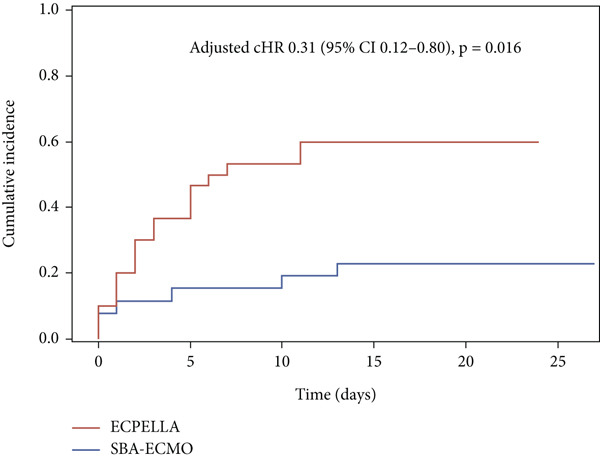
Cumulative incidence of serious bleeding. SBA‐ECMO, selective biatrial ECMO technique; ECPELLA, Impella CP/5.0 on top of ECMO; adjusted cHR, adjusted hazard ratio; CIs, confidence intervals.

Serious bleeding occurred in 26 (45.6%) patients (Table 2). The cumulative incidence curves of serious bleeding were different between the groups (Figure 3). SBA‐ECMO was associated with less serious bleeding than ECPELLA before (HR 0.28 [95% CI 0.11–0.71], *p* = 0.007) and after adjustment for confounders (adjusted cHR 0.31 [95% CI 0.12–0.80], *p* = 0.016).

### 3.4. Thrombotic Complications

**Figure 4:**
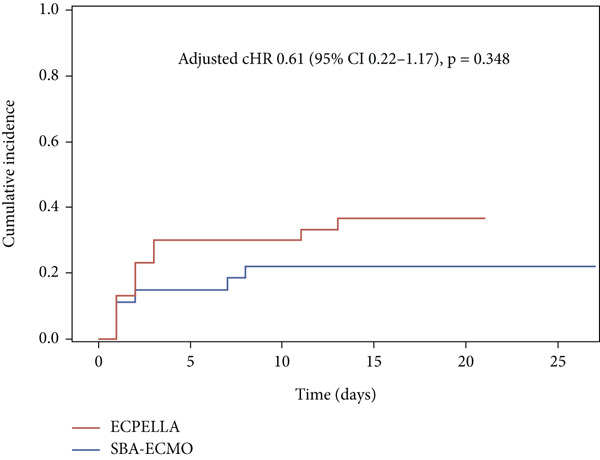
Cumulative incidence of thrombotic complications. SBA‐ECMO, selective biatrial ECMO; ECPELLA, Impella CP/5.0 on top of ECMO; adjusted cHR, adjusted cause‐specific hazard ratio; CIs, confidence intervals.

Thrombotic complications occurred in 17 (29.8%) patients. No significant difference was observed between the groups (Table 2 and Figure 4) either before (HR 0.58 [95% CI 0.22–1.59], *p* = 0.297) or after adjustment (adjusted cHR 0.61 [95% CI 0.22–1.17], *p* = 0.348).

### 3.5. Twenty‐Eight‐Day Mortality

The overall 28‐day mortality after LV unloading was 27 (47.4%). No difference was observed between groups (Table 2) in the univariable (OR 1.06 [95% CI 0.38–3.0], *p* = 0.91) or multivariable (OR 0.81 [95% CI 0.25–2.61], *p* = 0.72) analyses.

## 4. Discussion

**Figure 5:**
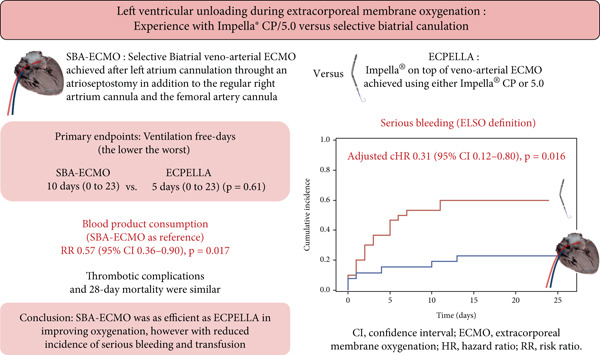
Central image summarizing main results. VA‐ECMO, venoarterial extracorporeal membrane oxygenation; LV, left ventricle; SBA‐ECMO, selective biatrial ECMO; ECPELLA, Impella CP/5.0 on top of ECMO.

To our knowledge, this study is the first to compare the performance of SBA‐ECMO with that of ECPELLA for LV unloading during adult VA‐ECMO support. The main findings were that SBA‐ECMO was associated with fewer bleeding complications and fewer transfusions than ECPELLA before and after adjustment for prespecified confounders (Figure 5).

In addition, although the number of VFDs was twice as high in the SBA‐ECMO group as in the ECPELLA group, the differences observed were not significant. This result may reflect the limited sample size of this study but underscores that SBA‐ECMO may be at least as effective as ECPELLA for alleviating LV distension and improving VFDs.

Previous data suggest differences in performance depending on the technique used [4, 16, 17], with several combinations of LV unloading studied. However, reports comparing IMPELLA with other preload LV unloading techniques are mostly case reports or case series with limited sample sizes [18, 19]. Their main information was that ECPELLA was not associated with better survival and that all preload unloading approaches were effective in reducing radiological signs of pulmonary edema.

To the best of our knowledge, a comparison between SBA‐ECMO and other LV unloading techniques is not available if SBA‐ECMO is carefully distinguished from PBAS without cannulation or left atrial VA‐ECMO. SBA‐ECMO offers several advantages over classical PBAS and left atrial VA‐ECMO. The presence of a cannula in each of the atria allows the level of drainage in each of the chambers to be tailored, with the possibility of selective flow monitoring on each venous line before the “Y” connector. Furthermore, the presence of a drainage cannula through the septum helps to avoid spontaneous closure of the PBAS performed during ECMO support.

The increased serious bleeding and transfusion risk associated with ECPELLA, despite a trend to a lower UFH exposition (Figure S2), may be explained by the biological abnormalities it may promote compared with SBA‐ECMO. In fact, SBA‐ECMO provides active drainage like that of ECPELLA, but unlike the latter, it may not increase the mechanical constraints and shear stress. In fact, the use of ECPELLA implies the addition of a pump to VA‐ECMO, with a high shear rate generated by each of the two pumps, the accumulation of which may further favor and worsen the severity of acquired von Willebrand syndrome [20], thrombopenia [21], and hemolysis.

Additionally, the SBA‐ECMO direct cost is less expensive and does not necessarily require an operating room for removal, unlike Impella 5.0 [22].

Interestingly, we observed no difference in thrombotic complications. This finding is in line with a recent study that reported no association between SBA‐ECMO and more ischemic or bleeding complications [14] compared to the control group. The rate of stroke in that study was 10% versus 9%, which is consistent with our own findings (Table 2) [23].

Our results underscore the safety profile of SBA‐ECMO compared to that of ECPELLA in terms of serious bleeding and BPC. However, SBA‐ECMO has a specific complication that needs to be considered, namely, the persistence of an atrial septal defect after cannula removal, which is estimated to occur in less than 10% of patients [14]. In our experience, most of these defects do not require percutaneous or surgical repair.

In this study, we focused on patients with LV congestion, where unloading is generally considered relevant. Despite our results, which appear to favor SBA‐ECMO, we must emphasize that neither approach is perfect and may have advantages in one clinical situation and disadvantages in another. This raises legitimate questions about the relevance of their routine prophylactic use in all patients, considering the currently available evidence.

In fact, the body of evidence supporting the survival benefit of systematic prophylactic unloading is limited. Well‐powered randomized clinical trials are lacking, and most studies are observational or retrospective. However, two meta‐analyses of these observational studies [6, 8] revealed improved survival with LV prophylactic unloading compared to no unloading, regardless of the type of unloading approach. For ECPELLA, relevant evidence is the large multicenter retrospective study with a propensity score–matched cohort, which showed improved survival but increased bleeding complications [9]. For SBA‐ECMO, the largest study available was retrospective, and the controls received ECMO support without LV unloading [12]. SBA‐ECMO reduced pulmonary congestion and achieved increased rates of successful ECMO weaning and heart transplantation [12]. However, this finding was challenged in an underpowered randomized controlled trial that revealed a reduction in pulmonary congestion but no improvement in the weaning rate or survival [24].

More recently, the focus has shifted to the timing of the initiation of LV unloading. A few studies have compared early initiation of LV unloading to more delayed initiation. One of those found an improved survival using ECPELLA [25]. However, the only available randomized controlled trial comparing early versus bailout unloading was underpowered, used SBA‐ECMO, and reported a shorter time to resolution of pulmonary congestion but no survival benefit [14].

This study has several strengths and limitations. The use of VFDs strengthened our results, as this variable integrates the duration of ventilation and survival [26]. This takes into account the competing risk between death, the duration of VA‐ECMO, and extubation, thus limiting misinterpretations that may arise from a crude comparison of ventilation duration or pulmonary congestion resolution, since, for example, ventilation duration would be shorter in a patient who dies on Day 1. However, respiratory improvement does not necessarily indicate the absence of persistent LV distension.

Alternative endpoints, such as changes on chest x‐rays to assess pulmonary edema, are limited by their modest diagnostic performance. In adult patients with high pretest probability of acute decompensated heart failure, a meta‐analysis of six prospective cohort studies reported a sensitivity of only 0.73 (95% CI 0.70–0.76) [27]. Similarly, the PaO_2_/FiO_2_ ratio may be an unreliable marker in this context, as it largely depends on the variable positioning of the mixing zone between native cardiac output and retrograde ECMO flow, as well as the arterial line location for blood sampling.

Another potentially interesting endpoint is the evolution of troponin levels; however, it may reflect the effect of coronary arteries revascularization and/or myocardial ischemia due to several causes beyond LV distension solely.

The other limits of our study are the observational and retrospective setting, which are prone to selection bias and confounders, despite the adjustments made using multivariable analyses. Despite being one of the largest available studies comparing LV preload unloading approaches, the sample size was relatively small. Therefore, this study seems underpowered to observed difference in some of the outcomes studied and is exposed to Type II errors. Indeed, difference in VFDs and thrombotic complications observed were nonsignificant. The lack of difference observed in the mortality rate is thus unconclusive. The lack of data about PCWP would have added more insight to our findings. Unfortunately, this variable was not available for analysis. However, PCWP may favor SBA‐ECMO, which generates suction directly in the left atrium contrarily to Impella. Furthermore, changes in PCWP may vary inversely with ECMO flow, decreasing in 36% of patients, remaining stable in 58%, and increasing in 6%. This variability makes it challenging to distinguish the effects of ventricular unloading from those of ECMO flow variations during support [28].

The ongoing randomized trials on LV unloading are all comparing ECMO with and without unloading using Impella (the REVERSE trial and the UNLOAD ECMO trial) and will provide additional information on routine LV unloading but not on SBA‐ECMO. Randomized trials are therefore still needed to compare the performance of these two approaches.

In conclusion, in patients with LV congestion with pulmonary edema during VA‐ECMO support, compared with ECPELLA, LV unloading with SBA‐ECMO was associated with fewer bleeding complications and transfusions despite similar VFDs, thrombotic complications, and mortality. Randomized trials are needed to confirm these findings.

**Nomenclature**
CAST: cannulated percutaneous atrioseptostomy
ECMO: extracorporeal membrane oxygenation
ELSO: Extracorporeal Life Support Organization
PBAS: percutaneous balloon atrioseptostomy
SBA‐ECMO: selective biatrial ECMO
VFDs: ventilation‐free days

## Disclosure

All authors contributed to manuscript reviewing and editing.

## Conflicts of Interest

The authors declare no conflicts of interest.

## Author Contributions

Conceptualization: M.D.M., J.R., V.L., A.V., and J.L. Data collection: J.R., M.D.M., V.L., L.T., O.L., L.L., and B.B. Methodology: M.D.M., A.V., M.J., N.Ro., A.M., F.J., and E.R. Data management: M.D.M., N.Ra., C.D., V.L., A.M., A.K., A.S.A.B., J.R., F.J., E.R., and B.B. Statistical analyses: J.L. and N.Ra. Original draft preparation: M.D.M., J.R., V.L., and M.J. M.D.M. and J.R. contributed equally to the study.

## Funding

No funding was received for this manuscript.

## Supporting information


**Supporting Information** Additional supporting information can be found online in the Supporting Information section. Figure S1: Chest rays showing in vivo positions of cannulas in biatrial ECMO and Impella on top of ECMO configurations. A1 shows the position of the drainage (venous) cannula in femorofemoral venoarterial ECMO support, without any unloading technique applied. B1 shows an additional drainage cannula (red arrows) inserted through the interatrial septum after percutaneous atrioseptostomy (canulated atrioseptostomy). This configuration allows selective biatrial venoarterial ECMO support. C1 shows an Impella CP device (yellow arrows) on top of femorofemoral venoarterial ECMO support. The ECMO venous cannula is indicated by the blue arrows. A2–C2 are processed from A1–C1, respectively, using contrast inversion to enhance cannula observation. Figure S2: Comparison of the daily maximum value of anti‐factor X activated values during the first 7 days of unloading. Tukey′s diagram showing the daily maximum values of anti‐factor X activated in ECPELLA patients (blue color) and in SBA‐ECMO patients in red. SBA‐ECMO, selective biatrial extracorporeal membrane oxygenation ECMO technique; ECPELLA, Impella CP/5.0 on top of ECMO.

## Data Availability

Data used for this study are available from the corresponding author upon a reasonable request that is in accordance with French and European Union data regulations.
