# Supplementary material for: Impella Versus Selective Biatrial Canulation for Left Ventricular Unloading During Extracorporeal Membrane Oxygenation
Source: Cardiovasc Ther. 2026 Feb 8;2026:3669575. doi: 10.1155/cdr/3669575 (PMC12883551; doi:10.1155/cdr/3669575)
Supplement: Supplementary file 1 — Supporting Information Additional supporting information can be found online in the Supporting Information section. Figure S1: Chest rays showing in vivo positions of cannulas in biatrial ECMO and Impella on top of ECMO configurations. A1 shows the position of the drainage (venous) cannula in femorofemoral venoarterial ECMO support, without any unloading technique applied. B1 shows an additional drainage cannula (red arrows) inserted through the interatrial septum after percutaneous atrioseptostomy (canulated atrioseptostomy). This configuration allows selective biatrial venoarterial ECMO support. C1 shows an Impella CP device (yellow arrows) on top of femorofemoral venoarterial ECMO support. The ECMO venous cannula is indicated by the blue arrows. A2–C2 are processed from A1–C1, respectively, using contrast inversion to enhance cannula observation. Figure S2: Comparison of the daily maximum value of anti‐factor X activated values during the first 7 days of unloading. Tukey′s diagram showing the daily maximum values of anti‐factor X activated in ECPELLA patients (blue color) and in SBA‐ECMO patients in red. SBA‐ECMO, selective biatrial extracorporeal membrane oxygenation ECMO technique; ECPELLA, Impella CP/5.0 on top of ECMO. [file CDR-2026-3669575-s001.docx]

**Left Ventricular Unloading during Extracorporeal Membrane Oxygenation: Experience with Impella CP/5.0 versus Selective Biatrial Canulation**

Roux J., Moussa D. M. et al.

**SUPPLEMENTAL MATERIAL**

**SUPPLEMENTAL METHODS**

**Description of the local unloading strategy for peripheral veno-arterial ECMO patients**

| **Before Impella use** | |
| --- | --- |
| First line | Intraaortic ballon pump  Cannulation change for right subclavian cannulation |
| Second line | - Left venting using - Right superior pulmonary vein cannulation through sternotomy in case of postcardiotomy etiology - Left superior pulmonary vein cannulation through left-sided thoracotomy - Apical cannulation in case of contraindications pulmonary vein cannulation |
|  | - Centralization in postcardiotomy patient and/or congenital heart disease |
|  | - Atrioseptostomy without cannulation |
| **Since Impella CP/5.0 introduction** | |
| First line | - Impella CP/5.0 - Cannulation change for right subclavian cannulation |
| Second line | - Left venting using - Left superior pulmonary vein cannulation through left-sided thoracotomy - Right superior pulmonary vein cannulation through sternotomy in case of postcardiotomy etiology - Apical cannulation in case of contraindications t pulmonary vein cannulation |
| Third line | - Centralization in postcardiotomy patient and/or congenital heart disease |
|  | - Intraaortic ballon pump |
|  | - Atrioseptostomy without cannulation |
| **Since cannulated atrioseptostomy introduction** | |
| First line | - Cannulated atrioseptostomy for percutaneous left atrial cannulation |
|  | - Impella CP/5.0 |
| Second line | - Left venting using   - Left superior pulmonary vein cannulation through left side thoracotomy   - Right superior pulmonary vein cannulation through sternotomy in case of postcardiotomy etiology   - Apical cannulation in case of contraindications to pulmonary vein cannulation |
| Third line | - Centralization for postcardiotomy patient and/or congenital heart disease |
|  | - Intraaortic ballon pump |
|  | - Atrioseptostomy without cannulation |
| –This left ventricle unloading policy procedure is specific to our center and is not intended as a recommendation. It considers local and national administrative particularities. In particular, the expertise available in each center for one or other of the technique, their costs and their reimbursement policy by the national health system.  –The choice of a device is a result of a joint decision by at least the attending intensivist and the cardiac surgeon. | |

**SUPPLEMENTAL FIGURES**

**Supplemental Figures legends**

**Additional Figure F1.**

A1 shows the position of the drainage (venous) cannula in femoro-femoral veno-arterial ECMO support, without any unloading technic applied. B1 shows an additional drainage cannula (Red arrows) inserted through the interatrial septum after percutaneous atrioseptostomy (canulated atrioseptostomy). This configuration allows selective biatrial veno-arterial ECMO support. C1 shows an Impella CP device (yellow arrows) on top of femoro-femoral veno-arterial ECMO support. The ECMO venous cannula is indicated by the blue arrows. A2, B2, C2, are processed from A1, B1 and C1 respectively, using contrast inversion to enhance cannulas observation.

## **Additional Figure F1. Chest rays showing in vivo positions of cannulas in biatrial ECMO and Impella on top of ECMO configurations**

**
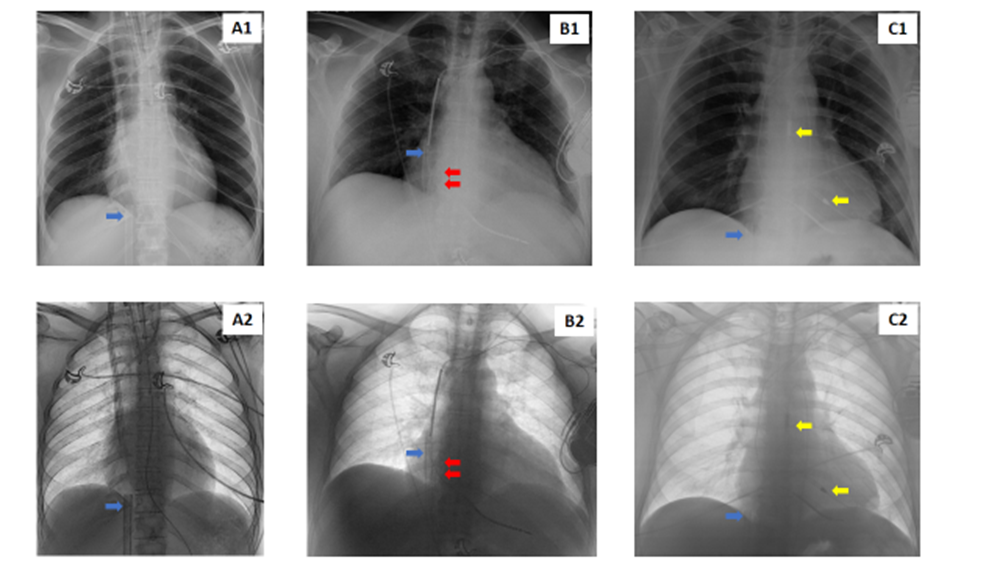
**

**Additional Figure F2. Comparison of daily maximum value of Anti-Factor X activated values during the first seven days of unloading**

**
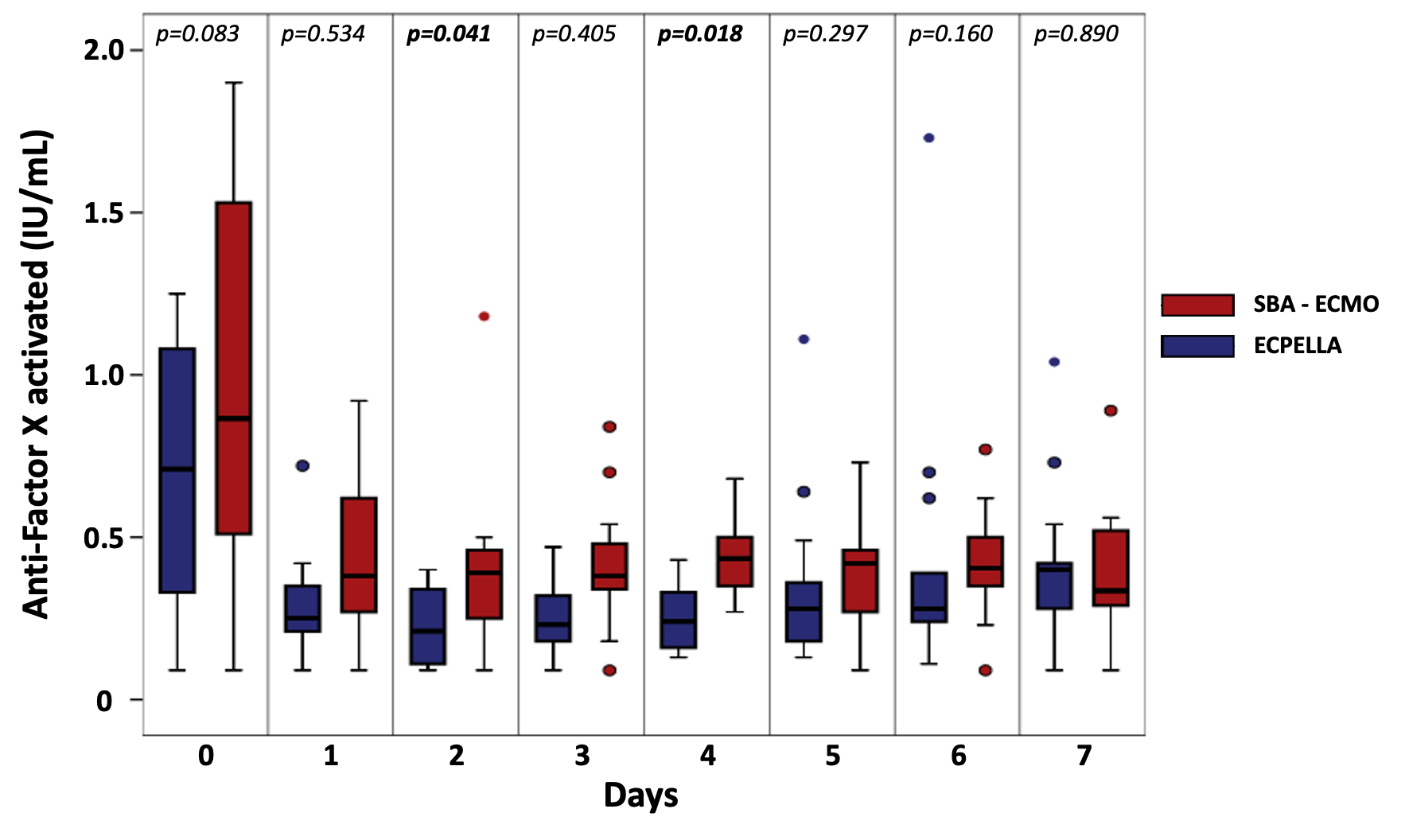
**
